# Allelic interaction effects of DNA damage and repair genes on the predisposition to age-related cataract

**DOI:** 10.1371/journal.pone.0184478

**Published:** 2018-04-24

**Authors:** Mei Yang, Junfang Zhang, Shu Su, Bai Qin, Lihua Kang, Rongrong Zhu, Huaijin Guan

**Affiliations:** Eye Institute, Affiliated Hospital of Nantong University, Nantong, Jiangsu Province, China; Justus Liebig Universitat Giessen, GERMANY

## Abstract

**Purpose:**

Age-related cataract (ARC) is a leading cause of visual impairment and blindness worldwide. DNA damage and malfunction of DNA repair are believed to contribute to the pathogenesis of ARC. Aside from increasing age, the risk factors for ARC appear to be rather complex, and one or more gene variations could play critical roles in the diverse processes of ARC progression. This study aimed to investigate the combined effects of different genetic variants on ARC risk.

**Methods:**

A cohort of 789 ARC patients and 531 normal controls from the Jiangsu Eye Study was included in this study. Genotyping of 18 single-nucleotide polymorphisms (SNPs) in 4 DNA damage/repair genes was performed using TaqMan SNP assays. SNP-SNP interactions were analyzed via multifactor dimensionality reduction (MDR), classification and regression tree (CART) and genetic risk score (GRS) analyses.

**Results:**

Based on single-locus analyses of the 18 SNPs examined, *WRN-rs11574311 (**T>C**)* was associated with ARC risk. However, in MDR, the gene-gene interaction among the five SNPs (*WRN-rs4733220 (**G>A**)*, *WRN-rs1801195 (**T>G**)*, *OGG1-rs2072668 (**G>C**)* and *OGG1-rs2304277 (**A>G**)*) on ARC risk was significant (OR = 5.03, 95% CI: 3.54~7.13). CART analyses also revealed that the combination of five SNPs above was the best polymorphic signature for discriminating between the cases and the controls. The overall odds ratio for CART ranged from 4.56 to 7.90 showing an incremental risk for ARC. This result indicated that these critical SNPs participate in complex interactions. The GRS results showed an increased risk for ARC among individuals with the SNPs in this polymorphic signature.

**Conclusion:**

The use of multifactorial analysis (or an integrated approach) rather than a single methodology could be an improved strategy for identifying complex gene interactions. The multifactorial approach used in this study has the potential to identify complex biological relationships among ARC-related genes and processes. This approach will lead to the discovery of novel biological information, ultimately improving ARC risk management.

## Introduction

Age-related cataract (ARC), a leading cause of visual impairment and blindness worldwide [1], is a growing global public health problem that affects approximately 37 million people and accounts for 48% of all cases of blindness[2, 3]. According to the location of the opacity within the lens, ARC can be classified as cortical (C), nuclear (N), posterior subcapsular (PSC) or mixed (M)[4]. The development of ARC can be influenced by multiple factors, ranging from degenerative processes or personal characteristics to environmental and dietary factors. Age, gender, smoking, and exposure to sunlight are the documented risk factors for ARC, and several recent studies have identified numerous single nucleotide polymorphisms (SNPs) in genes such as *OGG1*, *EPHA2*, *WRN* and glutathione S-transferases (*GSTs*) that are associated with ARC[5–8].

In the past, the majority of studies have analyzed individual genes by directly testing the effects of one or several SNPs in a candidate gene on disease development. However, because of the weak marginal effects of these disease-associated SNPs, each individual SNP has limited power to predict the risk of ARC. More recently, to evaluate whether interactions and combined effects among multiple SNPs contribute to the susceptibility to ARC, researchers have turned to multifactorial analysis. The analysis of such interactions and combined effects in case-control studies is hampered by one major concern: dimensionality.

In the current study, we employed a combination of three methods—multifactor dimensionality reduction (MDR), classification and regression tree (CART) analysis and genetic risk score (GRS) calculation—to extend our previous work[9] on ARC susceptibility by jointly investigating 18 SNP genotypes in 4 genes. This analytical approach avoided the problems related to dimensionality and multiple comparisons.

## Methods

### Study design and participants

The participants in this case-control study, including both ARC patients and normal controls, were recruited from the Jiangsu Eye Study (JES), a population-based epidemiologic study. We identified and selected ARC patients as research subjects from a total of 2208 cataract patients from the JES. According to the inclusion and exclusion criteria, 1064 ARC patients (C = 335, N = 470, PSC = 42, M = 217) were included. Applying further exclusion criteria led to the removal of 163 participants, including ARC patients with systemic diseases such as diabetes, kidney disease, or cancer and those with macular diseases or other retinal diseases, as well as 67 patients of any ARC subtype with LOCSIII grade<2 for the worse eye. As a result, 834 ARC patients were eligible for this study. Of these 834 ARC patients, 45 patients lacked samples for DNA extraction and genotyping. Ultimately, we examined 789 ARC patients. The details of the design and procedures of this study have been described elsewhere[9].

This study was conducted according to the Declaration of Helsinki and was approved by the Ethics Committee of the Affiliated Hospital of Nantong University. Each participant was fully informed of the purpose and procedures of the study and signed an Informed Consent Form.

### Selection of SNPs and genotyping

We selected haplotype-tagging SNP by searching Han Chinese data from the International HapMap Project (http://hapmap.ncbi.nlm.nih.gov/) using the Tagger program. The SNPs with a minor allele frequency (MAF)≥4% in the Hapmap CHB population were included. Further basic selection criterion was an r^2^ value≤0.8, excluding strong linkage disequilibrium between adjacent variants. Eighteen polymorphisms in *BLM (rs1063147*, *rs7183308*, *rs17273206*, *rs8027126*, *rs7175811*, *rs3815003*, and *rs6496724)*, *WRN (rs4733220*, *rs2725361*, *rs2725338*, *rs1801195*, *rs2725383*, *rs1863280*, and *rs11574311)*, *ERCC6 (rs4838519* and *rs4253038)* and *OGG1 (rs2072668* and *rs2304277)* were included in this study, and all non-binary polymorphisms were represented by a binary variable in the dominant model.

### Statistical analysis

Continuous variables were presented as means±standard deviation (SD) and were evaluated using t tests. Categorized variables were presented using numbers and percentages and were evaluated using the χ^2^ test or Fisher’s exact test. A two-tailed *P*-value of less than 0.05 was considered to be statistically significant. All statistical analyses were performed using SPSS software version 18.0 (SPSS, Inc., Chicago, IL, USA). Gene-gene interactions among the 18 loci of the 4 genes were determined using MDR (MDR software, version 2.0 beta8). A CART model was conducted using R (version 3.0.1) (http://www.rproject.org/).

### MDR

MDR is a non-parametric and genetic model-free method that uses constructive induction or attribute construction to identify the interactions among multi-locus genotypes. This method was initially introduced by Ritchie et al[10]. The benefit of MDR is the minimization of statistical issues such as invalidity of parameter estimates owing to the presence of few or no observations assigned to contingency table cells when testing for interactions. In MDR, a set of multi-locus genotypes with n dimensions is reduced to a single dimension (i.e. constructive induction) of 1 variable with 2 possible values for the genotypes of 2 loci: high risk or low risk. The model is evaluated for its ability to correctly classify and predict disease (case vs. control) according to the testing balanced accuracy (TBA) statistic[11], the cross-validation consistency (CVC) and permutation testing. One thousand permutations were repeatedly conducted for each randomized dataset to determine the statistical significance of the best models and to identify false positives. A *P*-value of less than 0.05 for the MDR permutation results was considered to be statistically significant.

### CART analysis

Decision trees date back to the early 1960s with the work of Morgan and Sonquist. Breiman and colleagues published the first comprehensive description of recursive partitioning methodology, a novel application of CART analysis to clinical and physiological data related to mood disorders, and this method therefore merits a more extensive description. CART is a powerful statistical method of data mining that can analyze data from different perspectives for summarization into useful and practical information in order to identify important correlations or patterns among dozens of variables in large relational databases. CART requires no distributional assumptions; however, CART models are highly unstable in response to small changes in the data, and this instability represents the major drawback of CART analysis. CART creates binary tree-shaped structures and classifies patients or predicts outcomes by selecting the most important genetic and environmental risk factors. The binary decision tree first considers all individuals pooled together in a heterogeneous root node. Before growing a tree, the measures for goodness of split are chosen using the criteria determined by the rpart package algorithm, which identifies splits that maximize the homogeneity of subnodes with respect to the value of the target variable. Each subnode can then be treated independently as a new root node, and the pattern continues until no further partitions can be performed, resulting in a very large and complex tree. In CART analysis, the primary variable used for splitting is examined together with other variables via a pruning procedure to avoid overfitting the model. Proportions closer to 0 or 1 are considered to reflect purer partitions. The binary tree structure shows the effects of interactions between variables using the optimal splits. Finally, the risk of various genotypes was evaluated using a special type of CART analysis: logistic regression analysis. However, for a large number of parameters, it is computationally infeasible to examine every possible combination of factors along with their interactions in a logistic regression. CART can precisely determine the combination of variables that maximizes predictive power.

### GRS

One popular approach of incorporating identified genetic variants is the calculation of a GRS for modeling using a variety of approaches, such as additive simple count and weighted GRSs[12, 13]. The applicability of these cumulative GRSs as predictive models for disease has been proposed and has shown anecdotal success in real genetic studies[14–18]. The GRS is defined as the cumulative number of risk-associated alleles in each individual. A value of 2, 1 or 0 was allotted to the homozygous variant, heterozygous and homozygous wild type genotypes, respectively, and the values for all 18 SNPs were then summed. We treated alleles with an odds ratio (OR) greater than or equal to 1 at each locus as a risk allele (the reference allele was considered as the risk allele if its OR was less than 1).

## Results

### Population characteristics

The demographic and clinical characteristics of the study subjects are presented in Table 1. A total of 1329 participants from the JES, including 789 ARC cases (C = 257, N = 366, PSC = 34, M = 132) and 531 controls, were recruited for the current study. The average age was 69.7 years for the cases and 70.4 years for the controls (*P* = 0.053). The gender distribution of the two groups was matched (*P* = 0.057). The fasting blood glucose level was not significantly different between the two groups (*P* = 0.063).

**Table 1:** Demographic characteristics of the study participants.

	Control (N = 531)		ARC (N = 789)		*P*
	Mean± SD	N(%)	Mean± SD	N(%)	
Age(years)	69.66±4.51		70.38±7.72		0.053
Gender					0.057
Male		240(45.2)		315(39.9)	
Female		291(54.8)		474(60.1)	
Fasting blood sugar	5.19± 0.88		5.28± 0.85		0.063

### Allelic distribution and associations of polymorphisms with ARC

Among the controls, the genotypes of all SNPs except for *BLM rs8027126* that were considered in the current study were in accordance with HWE. Thus, we excluded this SNP from our study.

We found that only WRN-rs11574311 (T>C) was associated with a statistically significantly increased risk for developing ARC (OR = 1.49, 95%CI: 1.17–1.90, P = 0.003), although its significance was attenuated after Bonferroni correction (P = 0.054; Table 2). The differences regarding the CC genotype of WRN rs11574311 between ARC and control were statistically significant (p = 0.005) in the genotype analysis. And in dominant model, it also indicated its harmful roles in developing ARC (OR = 1.55, P<0.05; Table 3)

**Table 2:** Allele distribution of SNPs in control and ARC subjects.

Gene name	SNPs Major/minor	Allele distribution Major/minor (%)		P	OR(95%CI)
		Control	All cases		
*BLM*	rs1063147 (C/T)	898/164 (84.6)	1312/266(83.1)	0.36	1.11(0.90–1.37)
	rs7183308 (A/G)	1010/52(95.1)	1489/89(94.4)	0.41	1.16(0.82–1.65)
	rs17273206 (G/A)	829/233(78.1)	1206/372(76.4)	0.33	1.10(0.91–1.32)
	rs8027126 (G/T)	978/84(92.1)	1445/133(91.6)	0.63	1.07(0.81–1.43)
	rs7175811 (G/A)	766/296(72.1)	1116/462(70.7)	0.43	1.07(0.90–1.27)
	rs3815003 (T/C)	762/300(71.8)	1101/477(69.8)	0.27	1.10(0.93–1.31)
	rs6496724 (A/C)	738/324(69.5)	1059/519(67.1)	0.20	1.12(0.94–1.32)
*WRN*	rs4733220 (G/A)	620/442(58.4)	955/623(60.5)	0.27	0.92(0.78–1.07)
	rs2725361 (G/A)	693/369(65.3)	988/590(62.6)	0.17	1.12(0.95–1.32)
	rs2725338 (G/A)	779/283(73.4)	1187/391(75.2)	0.30	0.91(0.76–1.08)
	rs1801195 (T/G)	686/376(64.6)	985/593(62.4)	0.26	1.10(0.93–1.29)
	rs2725383 (G/C)	945/117(89.0)	1363/215(86.4)	0.06	1.27(1.00–1.62)
	rs1863280 (T/G)	831/231(78.3)	1229/349(77.9)	0.82	1.02(0.85–1.23)
	rs11574311 (T/C)	950/112(89.5)	1342/236(85.0)	0.003 *	1.49(1.17–1.90)
*ERCC6*	rs4838519 (A/C)	539/523(50.8)	801/777(50.8)	0.99	1.00(0.86–1.17)
	rs4253038 (A/G)	730/332(68.7)	1057/521(67.0)	0.35	1.08(0.92–1.28)
*OGG1*	rs2072668 (G/C)	636/426(59.9)	978/600(62.0)	0.30	0.92(0.78–1.07)
	rs2304277 (A/G)	616/446(58.0)	960/618(60.8)	0.14	0.89(0.76–1.04)

**Table 3:** Genotype distribution of SNPs in control and ARC subjects.

Gene	SNPs Major/minor	Genotype distribution (%)			P	Dominant OR(95%CI)	Recessive OR(95%CI)
			Control	ARC			
*BLM*	rs1063147	CC	379(71.37)	544(68.95)	0.624	1.12 (0.88–1.43)	1.18 (0.58–2.42)
		TC	140(26.37)	224(28.39)			
		TT	12(2.26)	21(2.66)			
	rs7183308	AA	479(90.21)	702(88.97)	0.429	1.14 (0.79–1.64)	_
		GA	52(9.79)	85(10.77)			
		GG	0(0.00)	2(0.25)			
	rs17273206	GG	323(60.83)	463(58.68)	0.582	1.09 (0.87–1.37)	1.25 (0.76–2.07)
		AG	183(34.46)	280(35.49)			
		AA	25(4.71)	46(5.83)			
	rs7175811	GG	277(52.17)	401(50.82)	0.640	1.06 (0.85–1.32)	1.21 (0.81–1.79)
		AG	212(39.92)	314(39.80)			
		AA	42(7.91)	74(9.38)			
	rs3815003	TT	274(51.60)	383(48.54)	0.535	1.13 (0.90–1.41)	1.22 (0.76–1.67)
		CT	214(40.30)	335(42.46)			
		CC	43(8.10)	71(9.00)			
	rs6496724	AA	263(49.53)	361(45.75)	0.401	1.16 (0.93–1.45)	1.11 (0.78–1.57)
		CA	212(39.92)	337(42.71)			
		CC	56(10.55)	91(11.53)			
*WRN*	rs4733220	GG	190(35.78)	282(35.74)	0.088	1.00 (0.80–1.26)	0.73 (0.55–0.98)
		AG	240(45.20)	391(49.56)			
		AA	101(19.02)	116(14.70)			
	rs2725361	GG	227(42.75)	312(39.54)	0.386	1.14 (0.91–1.43)	1.20 (0.86–1.66)
		AG	239(45.01)	364(46.13)			
		AA	65(12.24)	113(14.32)			
	rs2725338	GG	290(54.61)	448(56.78)	0.491	0.92 (0.73–1.14)	0.79 (0.51–1.21)
		AG	199(37.48)	291(36.88)			
		AA	42(7.91)	50(6.34)			
	rs1801195	TT	227(42.75)	311(39.42)	0.479	1.15 (0.92–1.44)	1.09 (0.79–1.49)
		GT	232(43.69)	363(46.01)			
		GG	72(13.56)	115(14.58)			
	rs2725383	GG	422(79.47)	594(75.29)	0.148	1.27 (0.98–1.66)	1.70 (0.74–3.89)
		CG	101(19.02)	175(22.18)			
		CC	8(1.51)	20(2.53)			
	rs1863280	TT	326(61.39)	474(60.08)	0.718	1.06 (0.84–1.32)	0.87 (0.52–1.48)
		GT	179(33.71)	281(35.61)			
		GG	26(4.90)	34(4.31)			
	rs11574311	TT	428(80.60)	575(72.88)	0.005	1.55 (1.19–2.01)	1.66 (0.76–3.64)
		CT	94(17.70)	192(24.33)			
		CC	9(1.69)	22(2.79)			
*ERCC6*	rs4838519	AA	135(39.36)	208(60.64)	0.286	0.95 (0.74–1.22)	1.18 (0.92–1.53)
		CA	269(42.30)	367(57.70)			
		CC	127(37.24)	214(62.76)			
	rs4253038	AA	247(46.52)	359(45.50)	0.349	1.04 (0.84–1.30)	1.31 (0.91–1.90)
		GA	236(44.44)	339(42.97)			
		GG	48(9.04)	91(11.53)			
*OGG1*	rs2072668	GG	195(36.72)	308(15.08)	0.560	0.91 (0.72–1.14)	0.87 (0.65–1.17)
		GC	246(46.33)	362(45.88)			
		CC	90(16.95)	308(39.04)			
	rs2304277	AA	184(34.65)	295(37.39)	0.322	0.89 (0.71–1.12)	0.81 (0.61–1.09)
		AG	248(46.70)	370(46.89)			
		GG	99(18.64)	124(15.72)			

### Gene–gene interactions

We compared the allele frequency between ARC cases and controls and then analyzed the distribution of allele frequencies stratified according to ARC subtype.

### MDR analysis

Table 4 shows the best interaction model based on MDR analysis for all ARC cases and controls for the one-locus through five-locus models. The one-factor model for predicting ARC risk was *WRN-rs11574311 (**T>C**)* SNP (testing accuracy = 0.5378, CVC = 10/10, permutation test *P*<0.001). The two-factor model with a potential gene-gene interaction between *OGG1-rs2072668 (**G>C**)* and *OGG1-rs2304277 (**A>G**)* showed an improved testing accuracy of 0.6193 and an increased CVC (10/10) (permutation test *P*<0.001). A significant three-factor model including the *BLM-rs7183308 (**A>G**)*, *OGG1-rs2072668 (**G>C**)* and *OGG1-rs2304277 (**A>G**)* SNPs yielded a testing accuracy of 0.6199 and a CVC of 06/10 (permutation test *P*<0.001); this model also implicated potential gene-gene interactions among the three included SNPs. There was also a significant four-locus model of *ERCC6-rs4253038 (**G>C**)*, BLM-rs6496724 *(**A>C**)*, *OGG1-rs2072668 (**G>C**)* and *OGG1-rs2304277*, which showed an improved testing accuracy of 0.6266 (*P*<0.001). Based on the TBA statistic and the permutation test *P*-values, compared with the single-factor models, the multifactorial model that included *WRN-rs11574311 (**T>C**)*, *WRN-rs4733220 (**G>A**)*, *WRN-rs1801195 (**T>G**)*, *OGG1-rs2072668 (**G>C**)* and *OGG1-rs2304277 (**A>G**)* was regarded as the best fit model with the highest testing accuracy of 62.73%, the greatest CVC of 10/10, and a permutation test *P*-value<0.001. The results shown in Table 4 represent the associations of higher-order interactions with the risk of the C, N and M subtypes of ARC.

**Table 4:** Association of higher-order interactions with overall ARC risk based on MDR Analysis.

No. of interacting loci	Best Interaction Model	Testing Accuracy	CVC	*P* for 1000 permutation Testing
ALL				
1	*WRN-rs11574311*	0.5378	10/10	0.001
2	*OGG1-rs2072668*,*OGG1-rs2304277*	0.6193	10/10	<0.001
3	*WRN-rs4733220*,*OGG1-rs2072668*,*OGG1-rs2304277*	0.6199	6/10	<0.001
4	*ERCC6-rs4253038*,*BLM-rs6496724*,*OGG1-rs2072668*,*OGG1-rs2304277*	0.6266	6/10	<0.001
5	*WRN-rs11574311*,*WRN-rs4733220*,*WRN-rs1801195*,*OGG1-rs2072668*,*OGG1-rs2304277*	0.6273	10/10	<0.001
C				
1	*WRN-rs11574311*	0.5637	10/10	<0.001
2	*OGG1-rs2072668*,*OGG1-rs2304277*	0.6228	9/10	<0.001
3	*WRN-rs4733220*,*OGG1-rs2072668*,*OGG1-rs2304277*	0.6301	4/10	<0.001
4	*WRN-rs11574311*,*WRN-rs4733220*,*ERCC6-rs4838519*,*OGG1-rs2072668*	0.6397	5/10	<0.001
N				
1	*BLM-rs1063147*	0.5333	8/10	0.038
2	*OGG1-rs2072668*,*OGG1-rs2304277*	0.6157	10/10	<0.001
3	*WRN-rs2725361*,*OGG1-rs2072668*,*OGG1-rs2304277*	0.6287	9/10	<0.001
4	*WRN-rs4733220*, *BLM-rs6496724*,*OGG1-rs2072668*,*OGG1-rs2304277*	0.6042	4/10	<0.001
5	*BLM-rs1063147*,WRN-rs4733220,*WRN-rs2725361*,OGG1-rs2072668,OGG1-rs2304277	0.5595	7/10	0.01
M				
1	*WRN-rs11574311*	0.5817	7/10	0.001
2	*OGG1-rs2072668*,*OGG1-rs2304277*	0.6051	10/10	<0.001
3	*BLM-rs6496724*,*OGG1-rs2072668*,*OGG1-rs2304277*	0.6319	10/10	<0.001
4	*WRN-rs11574311*, *BLM-rs7175811*,*WRN-rs2725338*,*OGG1-rs2304277*	0.6292	6/10	<0.001

### CART analysis

The final tree structure, which contained nine terminal nodes, was generated via CART analysis for identification of ARC-related factors, considering all investigated genetic variants of the selected pathways (Table 5). CART analysis showed that patients with higher and lower risks of ARC could be identified based on specific genotype combinations. Consistent with the best one-factor MDR model, the initial factor splitting the root node on the decision tree was *WRN-rs11574311*; this result suggests that this SNP is the strongest risk factor for ARC among the polymorphisms examined. Thus, individuals carrying *WRN-rs11574311* were categorized as high risk for ARC with an OR of 7.90 (95%CI: 2.87–21.74, *P*<0.001). Using the terminal node that had the lowest percentage of cases (36.9%), representing the *WRN-rs11574311*, *WRN-rs4733220*, *WRN*-rs1801195, *OGG1-rs2304277* and *OGG1-rs2072668* genotypes, as a reference, the *P*-values of all subgroup combinations except the second, third and fourth combinations were statistically significant. The individuals carrying the combination of *WRN-rs11574311*, *WRN-rs4733220* and *BLM-rs17273206* exhibited a significantly increased risk for ARC (OR = 4.56, 95%CI: 1.58–13.18), and individuals carrying the combined genotypes of *WRN-rs11574311*, *WRN-rs4733220*, *WRN-rs1801195*, *OGG1-rs2304277* and *OGG1-rs2072668* also had increased risk for ARC. Thus, based on the single-locus analysis, *BLM* or *OGG1* may not be responsible for conferring high risk for ARC. However, the CART and MDR analyses suggest a higher-order gene-gene interaction between *BLM* and *OGG1*. The results shown in Tables 6, 7 and 8 represent the associations of higher-order interactions with the C, N and M subtypes of ARC risk, respectively. These results were consistent with those obtained from MDR analyses.

**Table 5:** Risk estimates of CART Terminal Nodes for all ARC patients.

Nodes	*WRN-rs11574311*	*WRN-rs4733220*	*BLM-rs17273206*	*WRN-rs1801195*	*WRN-rs1863280*	*OGG1-rs2304277*	*OGG1-rs2072668*	*P*	OR(95%CI)
1	TT	AG/GG	-	GT/TT	-	AA/AG	CC	-	Reference
2	TT	AG/GG	-	GT/TT	-	GG	GC/GG	0.76	1.22(0.34–4.32)
3	TT	AG/GG	-	GG	GT			0.45	1.63(0.46–5.72)
4	TT	AA	AG					0.14	2.36(0.75–7.43)
**5**	**TT**	**AA**	**AA/GG**					**0.003**	**4.56(1.58–13.18)**
**6**	**TT**	**AG/GG**	**-**	**GG**	**GG/TT**			**0.003**	**5.17(1.70–15.72)**
**7**	**TT**	**AG/GG**	**-**	**GT/TT**	**-**	**AA/AG**	**GC/GG**	**<0.001**	**6.42(2.37–17.44)**
**8**	**TT**	**AG/GG**	**-**	**GT/TT**	**-**	**GG**	**CC**	**<0.001**	**6.50(2.24–18.82)**
**9**	**CC/CT**							**<0.001**	**7.90(2.87–21.74)**
P trend								<0.001	

**Table 6:** Risk estimates of CART Terminal Nodes for patients with the C subtype of ARC.

Nodes	*WRN-rs11574311*	*ERCC6-rs4838519*	*WRN-rs4733220*	*P*	OR(95%CI)
1	CC/TT			-	Reference
2	CT	CA		0.23	1.34(0.83–2.16)
3	CT	AA/CC	AA/AG	<0.001	2.38(1.41–4.02)
4	CT	AA/CC	GG	<0.001	6.31(2.59–15.38)
P trend				<0.001	

**Table 7:** Risk estimates of CART Terminal Nodes for patients with the N subtype of ARC.

Nodes	*BLM-rs1063147*	*OGG1-rs2072668*	*BLM-rs6496724*	*WRN-rs2725361*	*WRN-rs4733220*	*P*	OR(95%CI)
1	CC	CC				-	Reference
2	TC/TT	-	AA			0.64	1.19(0.59–2.40)
3	TC/TT	-	CA/CC	AG/GG	AA/GG	0.26	1.41(0.78–2.54)
4	CC	GC/GG				0.07	1.55(0.97–2.48)
5	TC/TT	-	CA/CC	AG/GG	AG	<0.001	2.81(1.54–5.10)
6	TC/TT	-	CA/CC	AA		<0.001	4.88(1.97–12.06)
P trend						<0.001	

**Table 8:** Risk estimates of CART Terminal Nodes for patients with the M subtype of ARC.

Nodes	*WRN-rs11574311*	*WRN-rs2725338*	*BLM-rs7175811*	*WRN-rs4733220*	*P*	OR(95%CI)
1	CC/CT	GG	AG		-	Reference
2	TT				0.52	1.34(0.55–3.26)
3	CC/CT	GG	AA/GG	AA/AG	0.28	1.94(0.58–6.47)
4	CC/CT	GG	AA/GG	GG	0.001	5.00(1.82–13.70)
5	CC/CT	AA/AG			<0.001	8.57(2.32–31.72)
P trend					<0.001	

We also used logistic regression (LR) to detect SNP-SNP interactions from both MDR and CART and found that a p for interaction was 0.0627 using 5 SNPs in MDR model and was 0.9142 using 6 SNPs in CART model.

### GRS

Table 9 shows the additive effects of multiple SNPs. For each individual, we counted the number of alleles associated with increased risk for ARC. The total GRS ranged from 3 to 26 for all 1320 participants, with a median of 11 among control subjects and 12 among cases. The mean (±SD) total GRS was 12.08±4.07 in ARC patients and 11.46±3.77 in controls (*P* = 0.005). The patients with the C and N subtypes of ARC had higher total GRSs than the controls. We categorized the participants into 13 groups and considered participants with a GRS of 3–4 as the reference group. Compared with the reference group, the group of participants with a GRS<19 showed a non-significant difference in ARC risk. However, the significant OR (2.67, 95%CI: 1.08–6.66, *P* = 0.034) for the group of participants with a GRS>19 (19–26) compared with the reference group suggested that the former group had an increased risk of ARC. This result corresponded to a several-fold difference in ARC risk between those carrying lowest number of risk-associated alleles and those carrying the greatest number of risk-associated alleles in our population.

**Table 9:** Mean GRSs according to ARC subtype and corresponding P-values.

	Control	Case	*t*	*P*
All ARC	11.46±3.77	12.08±4.07	-2.80	0.005
C	11.46±3.77	12.14±4.01	-2.33	0.019
N	11.46±3.77	12.00±4.18	-2.03	0.042
M	11.46±3.77	12.17±3.81	-1.93	0.054

## Discussion

To more comprehensively evaluate ARC risk, the present analysis examined sets of sequence variants associated with high and low intrinsic risk of ARC. Of the 18 examined polymorphisms, several were found to be significantly associated with ARC risk in our previous study[9], while others showed little or no influence on the risk for ARC development. We took WRN-rs11574311 data as a reference for power analysis. Based on a pre-defined two-sided alpha of 0.05, our sample sets has greater than 85% power to detect a OR of 1.50. Moreover, accumulating evidence supports the importance of oxidative stress to ARC development, as oxidative stress induces various types of DNA damage in the lens, thus causing cataract[19–23]. Therefore, we further extended our work by incorporating factors potentially related to cataract pathogenesis. The genes selected in this study that encode for DNA repair enzymes play a vital role in the DSER, NER and BER pathways. To our knowledge, this is the first study to examine both main and epistatic effects of four candidate genes, *WRN*, *BLM*, *OGG1* and ERCC6, on the risk of ARC.

In the single-locus analysis, the *WRN-rs11574311 (**T>C**)* SNP was associated with ARC risk[9]. Furthermore, in the current study, we applied a multifactorial analysis strategy combining the MDR, CART and GRS approaches to systematically identify particular combinations of genetic variants that contribute to ARC risk. Therefore, a promising finding reported for the first time in this study was that the *WRN*, *BLM*, *OGG1* and ERCC6 genes may play an important role in independently modulating the etiology of ARC in an interactive manner.

Based on MDR analysis, the best 5-factor interaction model including *WRN-rs11574311 (**T>C**)*, *WRN-rs4733220 (**G>A**)*, *WRN-rs1801195 (**T>G**)*, *OGG1-rs2072668 (**G>C**)* and *OGG1-rs2304277 (**A>G**)* showed the highest testing accuracy compared with the single-factor models. Based on CART analysis, *WRN-rs11574311 (**T>C**)* polymorphism was also the most important individual susceptibility factor for ARC development. Moreover, gene-gene interaction analysis showed significant interactions among the *WRN-rs11574311 (**T>C**)*, *WRN-rs4733220 (**G>A**)*, *WRN-rs1801195 (**T>G**)*, *OGG1-rs2304277 (**A>G**)* and *OGG1-rs2072668 (**G>C**)* SNPs in association with ARC risk.

We used 10-fold cross-validation method to compare MDR and CART models and to evaluate if there is an over-fitting issue. In the 5-factor and their two-way interaction items model that was derived from MDR, the AUC from training data analysis was 0.6130, which was slightly higher than that from testing data analysis (AUC = 0.5566). The difference of these two AUCs was 0.0563. However, in the 7-factor and their two-way interaction items selected from Cart model the AUC from training data analysis 0.5703, which was slightly higher than that derived from testing AUC (0.5488, difference = 0.0215). AUC from model established by MDR is slightly high, compared with AUC from the model established by CART, which seems predictive performance of two models are similar in current study. There are smaller difference of AUCs between training data and testing data analysis in two models respectively, which implied that there is no serious over-fitting issue in both models.

The completed model increases with the order of interactions. Peduzzi P. et al. performed a Monte Carlo study and found that LR can detect only low-order interactions. This limitation of LR is referred to as the curse of dimensionality[24] and also consistent with what we found in this study. A fully saturated model with numerous terms may be prone to unstable and biased estimates due to sparse data and multicollinearity. In this condition, large sample theory underlying the test statistic may be violated. In some SNP-SNP interactions, Briollais et al. found that the permutation distribution of the likelihood ratio test did not closely match a chi square distribution, which justifies the use of a distribution-free test statistics[25]. CART and MDR do not require or assume any specific parametric or distributions for the relationship between predictors and outcomes. Therefore, they could uncover SNP-SNP interactions that are missed by LR. These model-free methods are better in dealing with sparse and high-dimension data and can account for non-linear SNP-SNP interactions. However, when the study design is suboptimal, such as relative small sample size or minor allele frequencies, these model-free methods have a high chance of detecting false associations[26].

Besides these, we also constructed and assessed a GRS from the number of risk alleles for risk assessment of ARC. We found individuals with ARC have a high GRS compared to normal individuals with the low GRS. Further calculating the GRS individually in the type of ARC, our finding was that all three types conferred increase risk.

Our multifactorial analytic approach revealed that the combination of the *WRN-rs11574311 (**T>C**)*, *WRN-rs4733220 (**G>A**)*, *WRN-rs1801195 (**T>G**)*, *OGG1-rs2304277 (**A>G**)* and *OGG1-rs2072668 (**G>C**)* SNPs may predicts a significantly increased risk for developing ARC. Furthermore, certain loci in the *WRN*, *OGG1*, *ERCC6* and *BLM* genes were associated with the C, N and M subtypes of ARC. Our number of included PSC cases remained small, even though we tried our best to increase the sample size, because the number of cases in this population-based study was fixed at the time point of the survey. The influence of genetic polymorphisms on the function of an enzyme may lead to different subtypes of cataract. Given the absence of cell nuclei in the lens nuclei, the functional effects of these SNPs might originate from lens epithelial cells. Aberrant metabolism of lens epithelial cells can easily cause dysfunction in the lens fibers.

Expression of the *BLM* gene is thought to increase in the S and G_2_ phases of the cell cycle as a result of crossing over during homologous recombination-mediated DNA repair events[27]. *BLM* assures genomic integrity through faithful chromosome segregation. Mutations in *BLM* deleting or altering its helicase motifs and disabling its 3'-5' helicase activity may induce Bloom syndrome. Furthermore, several studies reported that *BLM* influences the selection of the pathway for the repair of double-strand breaks in human chromosomes[28] and that polymorphisms in *BLM* are associated with colorectal cancer[29] and breast cancer[30]. Although ARC pathogenesis is different from that of cancers, these diseases might share pathways related to aging and genome instability. Our results show the association of specific gene-gene interactions with subtypes of ARC as well as overall ARC.

The *WRN* gene is responsible for maintaining the genome and serves as an important link between repair of defective DNA and processes related to aging[31]. Previous studies have reported associations between *WRN* polymorphisms and age-related diseases such as myocardial infarction[32] and type 2 diabetes mellitus[33]. Recently, an Israeli study found that the *WRN* C1367T (rs1346044) polymorphism is not linked to cataract among the elderly[21]. However, our results showed that *WRN*-rs11574311 is associated with the C and M subtypes of ARC and that *WRN*-rs2725338 is associated with the M subtype of ARC based on either single-factor or multifactorial analysis. Additionally, rs11574311 showed strong linkage disequilibrium with rs1346044. This inconsistency in results between studies may be due to the genetic heterogeneity between populations and the limited sample size in the Israeli study.

Mutations in *ERCC6* gene may lead to Cockayne syndrome, which often presents as severe cataract[34] and AMD[35, 36]. ARC and AMD, both of which are age-related eye diseases, may be caused by long-term UV radiation, oxidative damage, aging and a similar set of genetic factors. In our study, we found gene-gene interaction effect of *ERCC6* on the risk for the C subtype of ARC. *OGG1* is responsible for the removal of 8-oxoguanine, which is produced via the incorporation of 8-oxo-dGTP from the oxidation of dGTP by ROS during DNA replication in the BER pathway. We selected two new common SNPs in *OGG1* and found gene-gene interaction effects of these SNPs on not only subtypes of ARC but also overall ARC.

Compared to the results of single-locus analyses, the overall analysis of all 18 selected polymorphisms did not diminish either overall ARC or the various subtypes of ARC. Thus, we concluded that the application of this multifactorial analytical approach was more sensitive and accurate than single-factor approaches and showed reasonable power for identifying genes for disease risk prediction.

Moreover, a prominent, significant role of oxidative stress processes was elucidated based on the GCSs of selected pathways independently or in combination. Therefore, exhaustive multi-factorial analyses using approaches such as MDR, CART and GRS are well recognized methods in understanding complex traits, such as disease susceptibility, and the etiology of complex diseases.

In summary, the results from this comprehensive study using multi-factorial genetic analysis to determine risk factors for ARC development suggest that individuals with more genetic variations in oxidative stress pathway genes may elevate the risk for ARC. This finding confirms the importance of applying a multigenic pathway-based approach to disease risk assessment. This finding also indicates that the development of ARC involves complex genetic interactions and proceeds via different pathways depending on the specific genetic background of the individual. The present study provides evidence supporting the contribution of oxidative stress pathway genes, most importantly the interactions between the *WRN*, *BLM* and *OGG1* genes, to the risk for ARC.

Thus, our results support the concept that genetic polymorphisms can be used as predictors of ARC risk and that combined analysis of multiple polymorphisms may enables more delineation of risk groups. Thus, our results suggest the future direction of association studies. These results must be replicated in other ethnic groups, as our study included only Chinese individuals.

## Supporting information

S1 Table Genotype of 18 SNPs in control and ARC subjects. It contains the details of Genotype of 18 SNPs in control and ARC subjects and Cataract type of ARC subjects.
(XLSX)
